# Impact of Gastrointestinal Symptoms on Health-Related Quality of Life in an Australian Parkinson's Disease Cohort

**DOI:** 10.1155/2022/4053665

**Published:** 2022-11-25

**Authors:** Jade E. Kenna, Megan C. Bakeberg, Maddison Y. Abonnel, Frank L. Mastaglia, Ryan S. Anderton

**Affiliations:** ^1^School of Health Sciences and Physiotherapy, University of Notre Dame Australia, Fremantle, Western Australia, Australia; ^2^School of Medicine, University of Western Australia, Nedlands, Western Australia, Australia; ^3^Centre for Neuromuscular and Neurological Disorders, University of Western Australia, Nedlands, Western Australia, Australia; ^4^Perron Institute for Neurological and Translational Science, Nedlands, Western Australia, Australia; ^5^School of Medicine, University of Notre Dame Australia, Fremantle, Western Australia, Australia; ^6^Institute for Health Research, University of Notre Dame Australia, Fremantle, Western Australia, Australia

## Abstract

**Background:**

Gastrointestinal symptoms (GIS) in people with Parkinson's disease (PwP) are often underreported and may remain untreated. Constipation is a common nonmotor symptom that can adversely affect health-related quality of life (QoL); however, the impact of other GIS has not been adequately investigated.

**Objectives:**

To investigate the relationship between QoL and constipation using the Bristol Stool Chart, bowel movement frequency, and a perceived constipation measure; and to explore the relationship between QoL and other GIS in an Australian PD cohort.

**Methods:**

The impact of constipation and other GIS on QoL, as measured using the PDQ-39 scale, was assessed in a cohort of 144 (89 males, 55 females) clinic-attending PwP. Constipation was assessed using the Bristol Stool Chart as well as a composite constipation measure, and the Gastrointestinal Symptom Rating Scale (GSRS) was used to rate other GIS. Covariate corrected linear regression models were utilised to determine significant associations between GIS and QoL scores.

**Results:**

Individual and combined constipation measures were significantly associated with poorer QoL (*p*=0.032 and *p*=0.002, respectively). Analysis of GSRS symptom domains showed that in addition to symptoms of gastrointestinal hypomotility, a number of other symptoms such as increased eructation and increased flatus were also associated with poorer QoL.

**Conclusions:**

The findings point to the importance of GIS as contributor to health-related QoL in PwP. A better understanding of the relationship between GIS and QoL will help facilitate the development of more effective screening and treatment programs to improve symptom management and QoL for PwP.

## 1. Introduction

Parkinson's disease (PD) is a complex, progressive neurodegenerative disorder that was originally typified by its characteristic and clinically defining motor symptoms including, tremor, bradykinesia, rigidity, and postural instability. It is now recognised that a plethora of nonmotor symptoms (NMS) also occur and may antedate the characteristic motor symptoms of the disease [1–3]. These NMS include but are not limited to cognitive decline, mood and sleep disturbances, an impaired sense of smell, and a wide range of gastrointestinal symptoms [3, 4]. In fact, research indicates that the NMS of PD may have a greater impact on health-related quality of life than the cardinal motor symptoms [5, 6]. As PD does not have a cure or treatment to slow disease progression, research needs to focus on identifying factors that can be treated to improve patient quality of life.

Health-related quality of life (QoL) is defined as “the patient's own perception and self-evaluation regarding the effects of an illness and its consequences on his or her life” [7]. In complex, progressive disorders, like Parkinson's disease, with a wide range of motor and nonmotor symptoms, QoL is an important factor to consider when managing the disease as each individual will be unique, in terms of the combination of symptoms at any particular time-point in the course of the disease. Although constipation can affect QoL in PwP [6, 8, 9], gastrointestinal symptoms remain as the top underreported [10, 11], and thus, untreated symptoms in PD [5], leading to suboptimal clinical care and poorer QoL [12]. Whereas the adverse effects of other NMS such as cognitive impairment [13, 14] and sleep disturbances [15, 16], on QoL in PD are well-documented, the impact of gastrointestinal symptoms on QoL in PD populations has received relatively little attention in the literature.

Gastrointestinal symptoms (GIS), including constipation and nausea, are reported to be amongst the earliest symptoms to develop in PD, manifesting up to 20 years prior to the clinically defining motor symptoms [17, 18]. It is reported that 60–80% of people with PD (PwP) experience constipation [19], with almost all PwP experiencing GIS at some time throughout their disease course. A positive relationship also exists between inflammatory bowel diseases and the risk of developing PD and has been reported in many populations [20–22]. Additionally, proinflammatory bacterial species in the gut microbiome, which can manifest clinically as GIS, have been shown to be increased in PwP [23–28]. Thus, the link between GIS and PD is well established. As such, a disease-specific Gastrointestinal Dysfunction Scale for PwP has recently been proposed [29]. However, the extent to which GIS specifically impact on QoL in people with PwP remains unclear. Therefore, there is a need to better understand the relationship between constipation and other GIS and QoL in PwP to help facilitate the development of more effective screening and treatment programs and to improve patient quality of life.

The aims of the present study were: (i) to investigate the relationship between QoL and constipation using the Bristol Stool Chart, bowel movement frequency, and a perceived constipation measure; and (ii) to further explore the relationship between QoL and other GIS using the comprehensive Gastrointestinal Symptom Rating Scale (GSRS), in an Australian PD cohort.

## 2. Materials and Methods

### 2.1. Ethical Compliance

The study was approved by the Human Research Ethics Committees of The University of Western Australia (RA/4/20/4470) and St. Vincent's Hospital, Melbourne (LRR137/18). Prior to inclusion in the study, all participants were required to provide written informed consent and were free to withdraw from the study at any time. All work was conducted in accordance with the Declaration of Helsinki (1964).

### 2.2. Participants

Participants diagnosed with idiopathic PD (*n* = 144; 89 *M*/55 *F*) were recruited from Movement Disorders Clinics at the Perron Institute for Neurological and Translational Science (Perth, WA) and St. Vincent's Hospital (Melbourne, VIC) and assessed at respective clinics by the same researcher (JK) to mitigate potential bias. All participants were previously examined by a movement disorders neurologist for verification of the diagnosis in accordance with the UK Brain Bank Criteria for idiopathic PD [30], were ambulant, and none were known to have any other neurological disorders.

### 2.3. Clinical Assessments

Participants were assessed in the “ON” state using the Movement Disorders Society Unified Parkinson's Disease Rating Scale (MDS-UPDRS) Parts I–IV, with a higher score representing greater disease severity. Nonmotor and motor experiences of daily living were evaluated by MDS-UPDRS parts I and II, respectively. The severity of motor symptoms, and motor complications were evaluated by MDS-UPDRS Parts III and IV, respectively, and overall disease severity was rated using the Hoehn and Yahr scale. PD medications were categorised into six medication classes: levodopa (L-DOPA), dopamine agonists (DA), catechol-O-methyl transferase inhibitors (COMT-I) with levodopa, monoamine oxidase inhibitors (MAO-I), anticholinergics, and amantadine, and reported daily dosages were converted into a levodopa equivalent daily dosage (LEDD), as previously described [31]. Gastrointestinal symptoms were assessed using the Gastrointestinal Symptom Rating Scale (GSRS) as previously described [32]. The GSRS is a 15-questionpatient-compiled scale in which gastrointestinal symptoms during the preceding 3 months are evaluated individually on a 0–4 scale.

### 2.4. Constipation Assessments

This study used three individual measures and one combined measure of constipation. The first individual measure, the Bristol Stool Chart, is an extensively used stool form measurement tool with an ordinal scale of stool types ranging from Type 1 (hardest) to Type 7 (softest). Types 1 and 2 are considered abnormally hard (indicative of constipation), Types 3, 4, and 5 are considered normal, and Types 6 and 7 are considered abnormally loose. The second individual measure recorded was the frequency of bowel movements per day. The third individual measure was a perceived constipation question, where participants were asked whether they considered themselves to suffer from constipation or not and hereon will be referred to as “self-reported constipation.” As constipation refers to hard to pass and/or infrequently passed stool [33], all three individual constipation measures were then coded together, with any individual fitting the criteria for constipation in two or more of the individual constipation measures being coded as “constipated” in the combined measure of constipation.

### 2.5. Quality of Life Assessment

Participants completed a self-report39-question Parkinson's disease quality of life (PDQ-39) questionnaire to evaluate Parkinson's disease specific health-related quality of life over the previous month. The PDQ-39 is one of the most frequently adopted and highly recommended assessments for QoL in individuals with PD [25]. Each question was answered on a 5-stage scale, ranging from 0 = never, 1 = occasionally, 2 = sometimes, 3 = often, 4 = always/or cannot do at all. This questionnaire measures the following eight domains of quality of life: mobility, activities of daily living, emotional wellbeing, stigma, social support, cognition, communication, and bodily discomfort. Higher scores represented a lower quality of life.

### 2.6. Statistical Analyses

Statistical analyses were run on IBM SPSS (v. 28.0.1.0, IBM Corporation). Quantitative data were expressed as mean (SD), and categorical variables were expressed as *n* (%). All variables were assessed for normality using the Kolmogorov–Smirnov and returned significant values (*p* < 0.001). Differences in mean PDQ-39 total score, when grouped according to constipation and stool measures, were determined using the Mann–Whitney *U* tests, when grouped into binary variables, and Generalised Linear Models (GLMs), when kept as continuous variables, to correct for covariates. To further investigate associations between GSRS scores and the PDQ-39 total score, naïve and corrected GLMs were utilized. To assess for multicollinearity, the variance inflation factor (VIF) was calculated for all independent variables. For all reported models, VIF values were less than 2. A nominal *p* value of <0.05 was regarded as being statistically significant.

## 3. Results

### 3.1. Cohort Demographic and Clinical Data

Demographic and clinical assessment details for the PwP cohort are presented in Table 1. The average age of participants was 65.6 (SD = 9.19), and just over half were males (61.8%). PwP had a mean MDS-UPDRS III motor score of 21.81 (SD = 14.16) and had an average Hoehn and Yahr score of 2.03 (SD = 0.77). Of the participants, the majority were taking antiparkinsonian medications (95.9%), with an average LEDD of 816.44 (SD = 629.69), and the most commonly taken medication was levodopa (84.7%).

### 3.2. Poorer Quality of Life Correlates with NMS and Constipation

**Figure 1:**
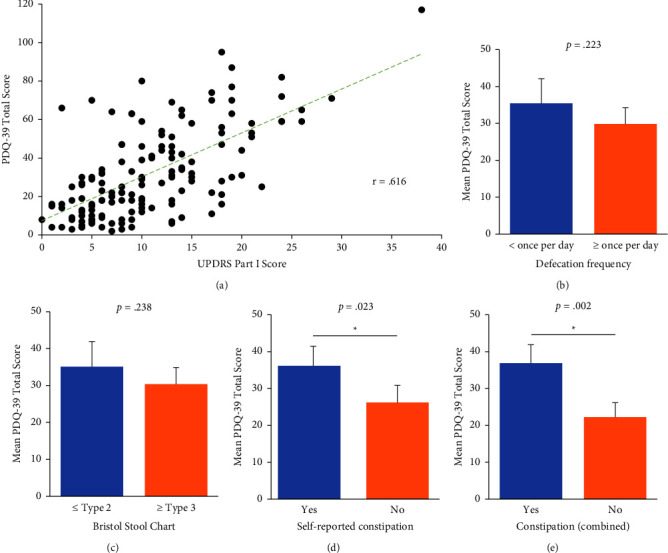
NMS and constipation measures' associations with PDQ-39 total scores in PD cohort. The association between PDQ-39 total scores and (a) UPDRS Part I, (b) Frequency of defecation, (c) Bristol stool chart stool rating, (d) Self-reported constipation question, and (e) all constipation measures (b)–(d) combined. Blue represents the constipated group and orange the not constipated group. *p* < 0.05 was considered statistically significant and indicated by^*∗*^.

Within the cohort, nonmotor symptoms of PD, measured by the UPDRS Part I, were significantly correlated with a poorer quality of life, as determined by the PDQ-39 score (*r* = 0.616, *p* < 0.001; Figure 1(a)). To determine the effect of constipation on QoL, the cohort was grouped based on multiple measures of constipation (Figure 1). When grouped by the frequency of bowel movements per day, with less than once a day grouped into the “constipated” group, and once or more into the “not constipated” group, individuals who passed less than one bowel movement a day trended towards a lower quality of life (*p*=0.223; Figure 1(b)). When grouped by the Bristol Stool Chart rating of their bowel movements, individuals who rated their bowel movements as Types 1 and 2 were grouped into the “constipated” group, and those who rated Types 3–7 into the “not constipated” group. Participants who were grouped into the “constipated” group also trended towards a poorer quality of life than those grouped into the “not constipated” group (*p*=0.238; Figure 1(c)). Participants were also asked whether they considered themselves to be constipated or not constipated in the self-reported constipation question, and those that considered themselves to be constipated had a significantly poorer quality of life than those who did not consider themselves constipated (*p*=0.023; Figure 1(d)). Lastly, when all measures of constipation were combined into one variable, those that were classed as “constipated” had a significantly poorer quality of life than those who were not classed as constipated (*p*=0.002; Figure 1(e)).

Univariate correlations showed significant associations between LEDD (*r* = 0.347, *p* < 0.001) and disease duration (*r* = .468, *p* < 0.001) with quality of life. As such, these factors were incorporated as covariates into GLM models looking at the above constipation measures (Table 2). GLMs revealed significant associations between the “self-reported constipation question,” and poorer quality of life in both naïve (*p*=0.005) and covariate corrected models (*p*=0.024). When all three constipation questions were combined, results also demonstrated a significant association with poorer quality of life in both naïve (*p* ≤ 0.001) and corrected models (*p*=0.006).

### 3.3. Poorer Quality of Life is Associated with GSRS Symptoms and Domains

Although the total gastrointestinal symptom score was not significantly associated with poorer quality of life, linear regression models showed that there was an association with specific gastrointestinal symptoms and symptom domains (Table 3). In the naïve models, increased eructation (*p*=0.027), decreased passage of stools (*p*=0.026), feeling of incomplete evacuation (*p*=0.002), and the hypoactive GI symptom domain (*p*=0.001) were all significantly associated with poorer QoL; while in the corrected models, increased flatus (*p*=0.011), and increased eructation (*p*=0.047) were also both significantly associated with poorer QoL.

## 4. Discussion

Previous QoL studies in Parkinson's disease have focused largely on the impact of NMS, such as sleep disturbances and cognitive impairment [13–16], rather than the range of gastrointestinal and other symptoms that PwP may experience. In the current study, we investigated various measures of constipation and their relationship with QoL in an Australian cohort of PwP and found that suffering from constipation was significantly associated with a reduced QoL as measured with the PDQ-39 instrument. We went on to further explore the relationship between other gastrointestinal symptoms and QoL using the comprehensive Gastrointestinal Symptom Rating Scale (GSRS), which has previously been used to evaluate the frequency and severity of gastrointestinal symptoms in assorted clinical settings [32, 34–45], but not in a QoL study in PwP. Our findings confirmed that, in addition to constipation and other symptoms of gastrointestinal hypomotility, a number of other gastrointestinal symptoms that are not included in current clinical assessment protocols also had a negative impact on QoL.

The UPDRS Part I is a measure of nonmotor activities of daily living and gives a broad overview of nonmotor symptoms experienced by the individual. As NMS are so prevalent in PwP, it is not surprising that we found a significant correlation between increasing difficulties with nonmotor symptoms and a poorer quality of life. These results confirmed similarly reported associations in various PwP populations where NMS were found to be a significant burden to QoL [1, 5, 9, 11, 13–16, 46, 47]. However, as the UPDRS Part I is very broad, it is also superficial in terms of measuring the burden of gastrointestinal symptoms, with only a single question relating to constipation, thereby overlooking the plethora of other gastrointestinal symptoms experienced by PwP. To this end, we therefore investigated the impact of constipation on the QoL of PwP using multiple measures.

The three individual constipation measures utilised in this study were, frequency of defecation, consistency of stool passed using the Bristol Stool Chart, and a question gauging whether participants considered themselves to be constipated or not (referred to as “self-reported constipation”). All three individual constipation measures were also grouped into one combined measure for constipation. All four measures revealed a trend towards constipation being associated with a poorer quality of life, with both the GSRS question and combined measures reaching statistical significance, even after adjusting for LEDD and disease duration. Although constipation is generally accepted as being one of the earliest prodromal symptoms in PwP [48], it is still considered a “taboo” topic by many individuals [11], and is thus less likely to be reported, and therefore, to remain untreated [5], and to significantly impact on QoL [12]. This is consistent with the findings of a study that reported a discrepancy of 76% between objectives versus subjectively reported constipation in a population of PwP [10].

In our study, we found that individual gastrointestinal symptoms, other than constipation also contributed significantly to a poorer QoL. These included increased eructation, decreased passage of stools, a feeling of incomplete evacuation, and increased flatus. The impact of these symptoms is compounded further by being more prevalent in PwP [32], contributing to an added burden on QoL that is disease specific. Thus, these results shed light on a group of symptoms that are being overlooked in relation to their contribution to QoL and that may be amenable to treatment in order to improve the QoL of PwP.

Parkinson's disease has been associated with a high burden of gastrointestinal dysfunction and comorbidities, in particular constipation and reduced colonic transit time, affecting up to 80% of all PwP [48, 49]. Furthermore, the severity of these GIS has been found to increase with the progression of the disease [50, 51]. Constipation, delayed gastric emptying, and changes in stool's consistency have also been linked with more rapid and severe motor and cognitive decline in PD [28, 32, 52]. In addition, an association between PD and inflammatory bowel diseases (e.g., Crohn's disease, ulcerative colitis, and irritable bowel syndrome) has been reported in assorted populations [22, 53]. Together, it is thought that, as a consequence of disease pathology, the function of the enteric nervous system can become progressively impaired in PwP [54–56]. This can result in impaired intestinal mobility and further exacerbation of gastrointestinal dysfunction as the disease progresses [57]. Thus, coinciding with evidence that gastrointestinal NMS can have considerable effects on the quality of life of PwP as well as placing a heavy burden on their carers [58].

## 5. Conclusions

In the present study, we have carried out an in-depth analysis of the frequency and impact of GIS on health-related QoL in a cohort of clinic-attending PwP. In addition to confirming the significant role of constipation and other symptoms of gastrointestinal hypomotility, our findings highlight a number of other GIS that are not commonly reported, that may also have significant effects on patient QoL. The results are consistent with the notion that current standard PD assessments do not comprehensively cover the full range of GIS [5, 32] and that individuals with PD are less likely to report GIS when not directly probed [11], meaning these symptoms are often overlooked, and may remain untreated, and contribute to poorer QoL. Thus, early identification and treatment of GIS in PwP has the potential to result in a significant improvement in QoL. Furthermore, it could help facilitate the development of more effective therapies to improve symptom management, and provide potential for developing a new diagnostic framework for recognising GIS in patients with early motor manifestations, or in the prodromal stages of the disease.

## Figures and Tables

**Table 1:** Demographic and clinical characteristics of the cohort.

Measures		PD (*n* = 144)
		Mean (SD) or *n*(%)
Age (years)		65.6 (9.19)
Gender	Male	89 (61.8%)
Age of onset		56.96 (10.56)
Disease duration		8.61 (6.02)
LEDD (mg)^^^		816.44 (629.69)
PD medications^^^	L-DOPA	122 (84.7%)
	DA	67 (46.5%)
	COMT-I	37 (25.7%)
	MAO-I	38 (26.4%)
	Amantadine	21 (14.6%)
	Anticholinergics	0 (0%)
	Unmedicated	6 (4.1%)
MDS-UPDRS	Part I	10.79 (6.63)
	Part III	21.81 (14.16)
Hoehn and Yahr		2.03 (0.77%)
	Stage 1	32 (22.2%)
	Stage 2	83 (57.6%)
	Stage 3	21 (14.6%)
	Stage 4	8 (5.6%)
	Stage 5	0 (0%)
History of GI illness		41 (28.1%)
	IBS	10 (6.9%)
	IBD	3 (2.05%)
	Other GI symptoms^#^	19 (13.0%)

^^^indicates two missing values among the PD cases. ^#^Other GI symptoms refers to other reported GI symptoms not organised into the 4 categories, such as diverticulitis, constipation, appendicitis, easily upset/sensitive stomach, recurring gastro episodes, strangulated bowel, incontinence. PD: Parkinson's disease. SD: standard deviation. LEDD: levodopa equivalent daily dosage. L-DOPA: levodopa. DA: dopamine agonists. COMT-I: catechol-O-methyltransferase inhibitor. MAO-I: monoamine oxidase inhibitor. MDS-UPDRS: movement disorders society unified Parkinson's disease rating scale. IBS: irritable bowel syndrome. IBD: inflammatory bowel disease. PU: peptic ulcer. GI: gastrointestinal.

**Table 2:** Analysis of associations between different measures of constipation and total PDQ-39 score in PD cohort.

Constipation measures	PDQ-39 total score					
	Naïve			Corrected^^^		
	Intercept	*β*-coefficient	*p* values	Intercept	*β*-coefficient	*p* values
Bristol stool chart	36.766	−1.55	0.288	10.719	1.326	0.318
Frequency of defecation	34.365	−2.98	0.146	15.573	0.043	0.981
Self-reported constipation	25.559	10.558	**0.005**	11.731	7.507	**0.024**
Constipation (combined)	22.208	14.375	**<0.001**	10.575	9.619	**0.006**

^^^corrected for LEDD and disease duration. PD: Parkinson's disease. PDQ-39: Parkinson's disease questionnaire—39. GSRS: Gastrointestinal Symptom Rating Scale.

**Table 3:** Analysis of associations between GSRS individual and total symptom scores and PDQ-39 score in PD cohort.

GSRS measures	PDQ-39 total score					
	Naïve			Corrected^^^		
	Intercept	*β*-coefficients	*p* values	Intercept	*β*-coefficients	*p* values
*General GI symptoms*						
Abdominal pain	31.730	0.245	0.942	15.645	−0.068	0.981
Borborygmus	32.488	−1.856	0.506	17.166	−3.592	0.140
Abdominal distention	30.876	1.75	0.463	16.011	−1.011	0.629
Increased flatus	33.113	−1.58	0.434	18.016	−4.487	0.011
Subdomain mean	32.212	−0.213	0.813	17.847	−1.374	0.079

*Upper GI symptoms*						
Heartburn	30.367	3.21	0.272	13.906	3.899	0.118
Acid reflux	31.353	0.799	0.762	15.487	0.312	0.893
Sucking sensation	31.804	−1.80	0.938	15.326	12.799	0.519
Nausea and vomiting	31.975	−0.852	0.833	15.132	1.723	0.621
Increased eructation	33.459	−7.27	0.027	17.422	−5.642	0.047
Subdomain mean	32.358	−0.392	0.769	15.296	0.218	0.850

*Hypoactive GI symptoms*						
Decreased passage of stools	28.979	6.13	0.026	15.374	1.588	0.528
Hard stools	28.465	3.30	0.087	16.022	−0.631	0.722
Feeling of incomplete evacuation	26.476	5.75	0.002	14.515	2.130	0.221
Subdomain mean	24.613	3.04	0.001	14.836	0.648	0.475

*Hyperactive GI symptoms*						
Increased passage of stools	32.341	−6.59	0.340	15.970	−2.544	0.670
Soft stools	31.435	1.71	0.615	14.060	4.770	0.103
Urgent need for defecation	32.384	−1.71	0.612	16.342	−2.305	0.426
Subdomain mean	32.195	−0.625	0.743	15.212	0.519	0.752

GSRS total score	27.941	0.589	0.222	16.770	−0.215	0.619

^^^corrected for LEDD and disease duration. GSRS: Gastrointestinal Symptom Rating Scale. PDQ-39: Parkinson's disease questionnaire—39. PD: Parkinson's disease. GI: gastrointestinal.

## Data Availability

The data are available upon reasonable request from the corresponding author.
